# Estimating treatment effects using parametric models as counter-factual evidence

**DOI:** 10.1186/s12874-025-02540-2

**Published:** 2025-04-09

**Authors:** Richard Jackson, Philip Johnson, Sarah Berhane, Ruwanthi Kolamunnage-Dona, David Hughes, Susanna Dodd, John Neoptolemos, Daniel Palmer, Trevor Cox

**Affiliations:** 1 University of Liverpool, Brownlow Hill, Liverpool, L69 3GL UK; 2 NIHR Birmingham Biomedical Research Centre, University Hospitals Birmingham NHS Foundation Trust and University of Birmingham, Birmingham, UK; 3 Institute of Applied Health Research, University of Birmingham, Birmingham, UK; 4 Department of General Surgery, University of Heidelberg, Im Neuenheimer Feld 420, 69120 Heidelberg, Baden-Württemberg Germany

**Keywords:** Causal Inference, Synthetic Controls, Efficacy, Parametric Models, Counter-Factual Evidence

## Abstract

Randomisation controlled trial are the gold standard for causal inference, however the rapidly increasing development of new treatments and the movement towards personalised medicine mean there is a need to measure efficacy outside of the costly and time-consuming RCT.

Here we propose a method of estimating treatment effects using parametric models to act as control against which to compare data from an experimental arm. This allows for treatment effects to be estimated where data are only available from an experimental arm and can be a tool useful in the analysis of observational cohorts or for the design and analysis of RCTs.

We develop this approach using Bayesian estimation procedures and compare this approach against other casual inference tools. We then demonstrate how this may be used to estimate the efficacy between two treatment in different RCTs for the analysis of Pancreatic Cancer.

It is proposed that with reasonable assumptions, this approach can produce a reliable estimate of efficacy and can have applications in both evaluating currently available data and in the efficient design of future trials.

**Supplementary Information:**

The online version contains supplementary material available at 10.1186/s12874-025-02540-2.

## Introduction

Randomised controlled trials (RCTs) are the gold standard method to performing causal inference [1] and whilst they are a robust experimental approach, they are also resource intensive, incurring large costs in terms of time, money and the number of patients required. In many cases RCTs are now considered to be unfeasible or even unethical [2]. These issues have contributed to the recommendation by the FDA for the use of real-world evidence [3] and externally controlled trials [4] in clinical research. The challenge for methodologists is to find efficient ways of estimating treatment effects outside the RCT framework.

The possibility of estimating causal effects from the growing availability of observational datasets is attractive and several causal tools have been developed to be applied in this setting. Multivariable modelling, instrumental variables [5], G-estimation [6] and Targeted Maximum Likelihood Estimators (TLME) [7] are example of possible approaches that have been applied, a review of which is offered by Nogueira et al. [8]. In clinical research, Propensity Score (PS) methods [9, 10] have become most prevalent having been applied in, for example, pancreatic cancer [11], Leukemia [12] and Alzheimer’s [13].

Whilst applied in different settings, these approaches aim to estimate an Average Treatment Effect (ATE) by accounting for potential confounders at a population level. Typically, datasets with patient level information on both the control and experimental groups are required.

Here we propose a method whereby a statistical model may be used as a control against which to compare the performance of an experimental treatment. Using this Model Estimated Controls (MEC) approach, for each patient exposed to an experimental therapy, a model is used to estimate their outcome had they received some control treatment. This generates patient level counter-factual evidence, and a comparison is then made between a patient’s observed response (under some experimental treatment) and their model-predicted counter-factual response to determine the efficacy of an experimental treatment.

This approach can be directly useful in several settings. Firstly, estimating treatment effects using observational data on experimental treatments and utilising the growing number of predictive models publicly available to act as a control. Secondly in the efficient design of single arm clinical trials where a statistical model can take the form of an external control arm and thirdly, in further evaluation of completed single-arm Phase II trials to estimate the potential efficacy of an experimental treatment.

Making use of modelling to derive efficacy whilst accounting for potential confounding is an approach that has been used in both G estimation and TMLE. Both these approaches estimate the expected outcome under each treatment group and then compare these expectations to derive an ATE. TMLE has the added aspect of using propensity scores to adjust these estimates and offers a means to directly obtain standard errors of the treatment effect. In doing so the TMLE is also ‘doubly robust’ ensuring that only one of the propensity score or model need be correctly specified to obtain a consistent estimator. Conceptually there is also some resemblance to Synthetic Controls [14] methodology as the counter factual evidence can be thought of as being synthetically generated although in practice there is little similarity in the procedures undertaken.

The approach suggested here differs both in its intended setting and in the procedures performed. Most notably whilst G-computation and TMLE typically compare the expected responses from each treatment group, the approach detailed here compares the observed response from an experimental group against the expected response of the control.

A method which allows for the estimation of treatment effects when data are available only on an experimental treatment provides an opportunity to evaluate treatments in a new setting. Taking as a starting point a data cohort of patients receiving some experimental treatment and a counter-factual model (CFM) predicting outcome under some control, we present non-parametric and likelihood-based methods which allow direct estimation of the target parameter and propose an estimation procedures to capture variability from both the CFM and the data cohort. We then compare this against other approaches used in similar settings and demonstrate its application to a real-world dataset.

The structure of this manuscript is as follows; Sect. 2 introduces the methodology and provides a motivating example in the setting of comparing data from a single arm early phase trial against an external model. Section 3 explores the method in a Causal framework and Sect. 4 provides a simulation study which compares the performance against G computation and Propensity Score weighting alternatives. Section 5 provides an example using real world data taken from 2 RCTs in pancreatic Cancer. Discussion is provided in Sect. 6.

## Methods and estimation

We begin with the premise that a data cohort are available on an experimental treatment only. The intention is to compare this against some statistical model which will be used to generate counter-factual evidence, hereafter referred to as the ‘Counterfactual Model’ (CFM). The working assumption is that for each patient in the data cohort, it is possible to predict their outcome under the control treatment using the CFM.

### Motivating example

Consider 10 patients from an early phase single arm clinical trial where we wish to estimate the potential efficacy of the experimental drug against some external control characterised by a parametric model.

Assume a treatment indicator Z=(0,1) and a binary outcome for the patients with potential outcomes Y(Z). As all patients in the study receive the experimental therapy, we observe Y(1). Further assume a single predictive covariate W. The data for the experimental arm consist of $\:({y}_{i},{w}_{i}$). The model to predict patients’ response to the control treatment (CFM) takes the form of a logistic regression model defined by

$$\:logit\left({s}_{i}\right)=\:{\eta\:}_{i}=a\:+b{w}_{i}$$

Where $\:{s}_{i}$ is the response. Assume the model is known with a = 0.037 and b = 0.395. From this, estimates of the linear predictor are obtained and used to estimate the expectation of an event under the control treatment conditional on the predictive covariate W, defined as

$$\:\psi\:\left(0\right|W)\:=\:Pr(Y\left(0\right)=1\left|W\right)$$

The data for this example are given in Table 1. Also provided are the observed outcome for the experimental treatment $\:\left[Y\right(1)$] and the difference between the observed and expected outcomes for each patient given by

**Table 1:** Example data to illustrate estimate of treatment effect to compare observed data against a parametric model

ID	$\:{w}_{i}$	$\:{\Gamma\:}_{i}$	$\:\psi\:\left(0\right)$	Y(1)	$\:\delta\:$
1	0.73	0.326	0.581	0	-0.581
2	0.43	0.207	0.552	1	0.448
3	0.38	0.187	0.547	1	0.453
4	0.30	0.156	0.539	0	-0.539
5	0.42	0.203	0.551	0	-0.551
6	1.16	0.496	0.621	1	0.379
7	1.76	0.733	0.675	1	0.325
8	1.76	0.733	0.675	0	-0.675
9	0.77	0.342	0.585	1	0.415
10	0.04	0.053	0.513	0	-0.513

$$\:\delta\:\:=\:Y\left(1\right)\:-\:\psi\:\left(0\right|W)$$

The aim to estimate an efficacy parameter, $\:\beta\:$, which measures the distance between the observed data and the model estimate. We propose two approaches to estimation. Firstly by directly sampling ‘control’ data from the CFM. Here the sampled control and experimental data can be directly compared as part of a simulated RCT. A second approach is proposed by defining a likelihood which includes an efficacy parameter $\:\beta\:$ which is obtained by extending the linear predictor to

$$\:logit\left\{{Y}_{i}\left(1\right)\right\}=\:{{\eta\:}_{i}}+\beta\:.$$

In the simple example illustrated in Table 1 we can use standard minimisation procedures to achieve $\:\beta\:$=-0.33 (and consequently and odds ratio comparing the experimental treatment to the control of exp($\:\beta\:)$= 0.71). In practice however, the parameters that describe the model (a & b above) are not fixed and will have their own source of error. Using a direct approach to obtain precision estimates about $\:\beta\:$ are therefore not appropriate. Section 2.2 and 2.3 detail a general likelihood and Bayesian estimation procedure which allow for error inherent in the CFM coefficients to be accounted for.

### Likelihood definition

The procedure proposed here is to define a likelihood which measures the observable difference between the data cohort and the CFM. The form of this likelihood depends on the form of the underlying model. Code has been developed in R for parametric survival models and any model which can be expressed as a member of the exponential family. This package is available from the GitHub repository RichJJackson/psc which includes further information on the derivation and application for each outcome type.

Here we provide an overview of the general likelihood formulation for outcomes obtained from generalised linear model and those obtained from parametric survival models. In doing this we obtain a general structure that can be applied in a wide number of scenarios.

Assume a fully parametric CFM is available defined by $\:\Phi\:(\Lambda\:,\gamma\:)$. Here $\:\Lambda\:$ are parameters which are common to all patients (e.g. model intercept) and $\:\gamma\:$ are the parameters associated with patient covariates. From the data cohort, assume that outcome data (y) and covariates (x) are available such that $\:D=({y}_{i},{\vec{x}}_{{i}})$, with $\:i=1,\ldots,N$ where $\:N$ is the number of individuals in the dataset. Note the set of covariates $\:{\vec{x}}_{{i}}$, directly relate to $\:\gamma\:$ in the CFM.

Using the information derived from the CFM, we first define $\:{\Gamma\:}_{i}$ which is a combination of the linear predictor ($\:{\upeta\:})\:$ and an efficacy parameter $\:\beta\:$ such that:

$$\:{\Gamma\:}_{i}=\:\gamma\:{\vec{x}}_{i}+\beta\:$$

#### Generalised linear models

Where the CFM, takes the form of a generalised linear model, some link function, $\:\text{G}(.)$ will also be specified to relate the linear predictor back to the scale on which the data are measured. We take advantage of the fact the GLM outcomes will follow the exponential family and specify a likelihood

$$\:L\left({y}_{i}\right|{\Gamma}_{i})=\prod_{i=1}^{N}\text{e}\text{x}\text{p}[{\text{y}}_{i}\theta\:-b\left(\theta\:\right)-c({\text{y}}_{i}\left)\right]$$

Where b(.), and c(.) represent the functions of the exponential family and $\:\theta\:$ is obtained as the inverse link function of the linear predictor $\:{\Gamma\:}_{i}$. Note that as a function of only the outcome, c(.), is not required for estimation.

Taking as an example a CFM fit using a binomial function with a logistic link function define $\:\theta\:={G}^{-1}\left({\Gamma\:}_{i}\right)\:$ where $\:{G}^{-1}\left(.\right)\:$ is the inverse logit function and $\:b\left(\theta\:\right)=\:\text{l}\text{o}\text{g}(1+\text{e}\text{x}\text{p}(\theta\:)$ ). A full likelihood is then

$$\:L\left({y}_{i}\right|{\Gamma}_{i})=\prod_{i=1}^{N}\text{e}\text{x}\text{p}\{{\text{y}}_{i}{G}^{-1}\left({\Gamma\:}_{i}\right)\:-n\:\text{l}\text{o}\text{g}[1+\text{exp}\left({G}^{-1}\left({\Gamma}_{i}\right)\right)\left]\right\}$$

#### Parametric survival models

With a time-to-event outcome, define $\:(\Lambda\:,\gamma\:)$ as a fully parametric model where $\:\Lambda\:$ are parameters associated with the estimation of the cumulative baseline hazard function. For the data cohort, assume data $\:D({t}_{i},{c}_{i},{\vec{x}}_{i})$, where the outcome data take the form of an event time $\:t$ and a censoring indicator $\:c$. Defining $\:{\Gamma\:}_{i}$ as above, the efficacy parameter ($\:\beta\:)$ now takes the form of a log hazard ratio.

A hazard function, $\:\text{h}\left({t}_{i}\right|\Lambda\:)$, and cumulative hazard function, $\:\text{H}\left({t}_{i}|\Lambda\:\right)$, are obtained from $\:\Lambda\:$ with its exact form depending on the parametric form of the hazard function in the CFM. Under a flexible parametric model [15] with a single internal knot define $\:\Lambda\:=({\eta\:}_{0},\:{\eta\:}_{1},{\eta\:}_{2})$ where knots are given by ($\:{\lambda\:}_{1},{\lambda\:}_{2},{\lambda\:}_{3})$. We then obtain

$$\:H\left({t}_{i}\right|\Lambda\:)={\eta\:}_{0}+{\eta\:}_{1}log({t}_{i})+{\eta\:}_{2}{z}_{2}$$

where

$$\:{z}_{2}=\left(log\right({t}_{i})-{\lambda\:}_{1}{)}_{+}^{3}+\xi\:(log\left({t}_{i}\right)-{\lambda\:}_{2}{)}_{+}^{3}+(1-\xi\:)\left(log\right({t}_{i})-{\lambda\:}_{3}{)}_{+}^{3}$$

The $\:{}_{+}$ subscript denotes negative values of a function being replaced by zero. From this, the hazard function is $\:h\left({t}_{i}\right|\Lambda\:)=\frac{\delta\:H\left({t}_{i}\right|\Lambda\:)}{\delta\:t}$, and so

$$\begin{array}{l}\:h({t_i}|\Lambda \:) = \frac{1}{t}\{ \eta {\:_1} + 3\eta {\:_2}[(log\left( {{t_i}} \right) - \lambda {\:_1})_ + ^2 + \\\xi \:(log({t_i}) - \lambda {\:_2})_ + ^2 + (1 - \xi \:)(log\left( {{t_i}} \right) - \lambda {\:_3})_ + ^2]\} \end{array}$$

It is straight forward to define $\:H\left(t|\Lambda\:,{\Gamma}_{i}\right)=\:{\Gamma\:}_{i}\:H\left({t}_{i}\right|\Lambda\:)$ and likewise for $\:h\left({t}_{i}|\Lambda\:,{\Gamma\:}_{i}\right).\:\:$ Estimates of the survival function and density function are obtained as $\:\text{S}\left({t}_{i}\right|\Lambda,{\Gamma}_{i})=\text{exp}\{-\text{H}\left({t}_{i}\right|\Lambda,{\Gamma}_{i}\left)\right\}$ and $\:\text{f}\left({t}_{i}|\Lambda\:,{\Gamma}_{i}\right)=-S\left({t}_{i}\right|\Lambda,{\Gamma}_{i}\left)\text{h}\right({t}_{i}|\Lambda,{\Gamma}_{i})$ respectively. A likelihood to evaluate survival outcomes can then be specified as

$$\:L\left(D\right|\Lambda,{\Gamma}_{i})=\prod\:_{i=1}^{N}\text{f}({t}_{i}|\Lambda,{\Gamma}_{i}{)}^{{c}_{i}}\text{S}({t}_{i}{|\Lambda,{\Gamma}_{i})}^{(1-{c}_{i})}.$$

Discussion on the use of $\:\beta\:$ as a valid tool for causal inference is included in Sect. 6.

### Estimation

The likelihood approach defined can be directly estimated using standard optimization (e.g. optim in R) routines to provide an estimate of $\:\beta\:$. However, this approach treats the CFM as a fixed entity whereas, in reality, the parameters which describe CFM will have their own source of error and correlation which should be accounted for. Estimation of $\:\beta\:$ ignoring these sources of error results in false levels of precision represented by confidence intervals for $\:\beta\:$ which have poor coverage.

We detail two estimation approaches; firstly an approach based on simulating data from the CFM to compare against data cohort as part of a simulated RCT and secondly estimating $\:\beta\:$ from the likelihoods as defined in Sect. 2.2.

For each approach to account for the inherent error of the CFM, the first step is to first draw a posterior sample from the CFM. Define the set of model parameters $\:B$ which summarize the parameters of the CFM ($\:\Lambda\:$ and $\:\gamma\:$ above). Let $\:\pi\:\left(B\right)$ to be the distribution of model parameters. Typically $\:\pi\:\left(B\right)\sim\:MVN(\mu\:,\Sigma\:)$ where $\:\mu\:$ is the vector of coefficient estimates from the CFM and $\:\Sigma\:$ is the variance-covariance matrix. Lastly define b as a single draw from $\:\pi\:\left(B\right).$

For the simulated RCT approach, use the sampled model, $\:\Phi\:\left(b\right)$, to generate random counterfactual outcomes for each observation in the data cohort. Note here the sampled outcome data will be taken from the conditional distribution of the outcome given the covariates included in the CFM. For each patient here a counter-factual control observation is generated resulting in 2N observations which can be directly compared as part of simulated RCT. The direct nature of the comparison will depend on the form of the outcome and the estimand required.

For the likelihood based approach, we define $\:{\gamma\:}_{i}$ as the combination of the linear predictor based on $\:b$ and the efficacy parameter $\:\beta\:$ (analogous to $\:{\Gamma\:}_{i}$ above). Define the likelihood $\:L\left(D\right|{\gamma\:}_{i})$ and estimate the posterior for $\:\beta\:$ as

$$\:P\left(\beta\:|b,D\right)\propto\:L\left(D\right|{\gamma\:}_{i},\beta\:\left)\:\pi\:\right(\beta\:)$$

For both approaches, we obtain an estimate of efficacy which are conditional on $\:b$. The marginal effects $\:\beta\:$ is obtained by resampling from $\:\pi\:\left(B\right)$. An MCMC based algorithm [16] is included in the supplementary material. This resampling process provides both the point estimate and variance of the efficacy between the control and experimental treatments. A comparison of both estimation methods is also provided in the supplementary material.

## Causal inference framework

Thus far, the mechanics of measuring the difference between a data cohort against a CFM have been proposed. Here it is discussed how this can be evaluating in the causal inference framework using the potential outcome notation of Imbens and Rubin [17].

Initially, consider a cohort of patients indexed by i = 1,…,N who are exposed to one of two possible treatments: Z ∈ (0,1). The potential outcomes for each patient under each treatment are expressed as random variables Y_i_(0) and Y_i_(1). Given only Y_i_(0) or Y_i_(1) can be observed, inferences are typically drawn on population averaged causal estimand

$$\:\partial\:=\:\frac{1}{N}\sum\:_{i=1}^{N}{\tau\:}_{i} $$

Where $\:{\tau\:}_{i}$ can be defined as the individual treatment effect which is not observed in practice. In this instance $\:\partial\:$ gives the Average Treatment Effect (ATE) which is reliably estimated when two groups are balanced, for example through randomization. More recently the development of causal inference tools has focused on the Conditional Average Treatment Effect (CATE) which can be defined as

$$\:{\tau\:}_{i}\left(x\right)=E[{Y}_{i}\left(1\right)-{Y}_{i}(O\left)\right|X={x}_{i}]$$

With a set of prognostic covariates X. The difference between two conditional estimates can then be expressed as

1 $$\:{\tau\:}_{i}\left(x\right)={m}_{1i}\left(x\right)-\:{m}_{0i}\left(x\right)$$

where

$$\:{m}_{1i}\left(x\right)=E\left[{Y}_{i}\left(1\right)\right|X={x}_{i}]$$

And equivalently for $\:{m}_{0}\left(x\right)$. Please note that other estimand (e.g. hazard ratios, odds ratios) may be obtained by altering the form of (1). As is general for causal inference methods, we require the assumption of strong ignorability, namely that the potential outcomes remain independent of the treatment approach conditional on X.

$$\:Y\left(1\right),Y\left(O\right)\perp\:Z|X$$

This allows us to define

$$\:{m}_{1i}\left(x\right)=E\left[{Y}_{i}\right(1\left)\right|X={x}_{i},Z=1]$$

Assuming consistency, the potential outcome, $\:{Y}_{i}\left(1\right),\:$ can be replaced with the observed outcome Y. The process of obtaining casual estimates via the comparison of two conditional expectations is well established. Using the approach detailed here, consider the scenario where the observed data consist entirely of patients on some experimental arm. Here then, $\:{m}_{1i}\left(x\right)$ can be defined directly from the data. We propose to generate counter factual evidence from a parametric model for each patient and define

$$\:{m}_{0i}\left(x\right)=E\left[{Y}_{i}\left(O\right)|X={x}_{i},Z=1\right]\:=\:f({x}_{i},Z=1)$$

Where f is then some function of the data x, here represented by our Counter-Factual Models. This allows for an estimand to be defined as

$$\:{\tau\:}_{i}\left(x\right)={m}_{1i}\left(x\right)-\:f({x}_{i},Z=1)$$

As for each individual both the observed and predicted outcomes are available, this theoretically allows for the direct estimation of the individual treatment effect although whether these estimates are reliable depends on the strength of the assumptions that are made. As both components of this estimand are conditional on Z = 1 this estimand is closest to being the Average Treatment Effect of the Treated (ATT) although under the assumption of exchangeability we can also use this to estimate the ATE. Treatment effect estimation can then be obtained by taking the expectation over the full population or restricting to some sub-population to give the conditional average treatment effect.

A key assumption required is that of no unmeasured confounders along with the need to assume that a patient’s potential outcome is not altered by the setting in which it is observed (the Stable Unit Treatment Variability Assumption). Given this method depends on a parametric model to estimate the counter factual evidence we also need assumptions that the model is correctly specified and that the posterior distribution of the model can be approximated by a multivariable normal distribution as used in the estimation procedure.

Importantly we also need to consider the assumptions of transportability and overlap. These state respectively that is first reasonable to apply the results of the CFM to the setting in which the data cohort were collected and that for every patient who received the experimental treatment, there exists some non-zero probability that they may have received the control treatment. Further it should be ensured that Inference is not performed beyond the supported range on which the model was constructed (e.g. we are not extrapolating beyond the intended setting).

## Simulation study

A simulation study is performed following the methods of Burton et al. [18] to demonstrate that firstly the efficacy parameter can be reliably estimated in several settings and secondly to provide some illustrative comparison against other available methodologies. To conduct the simulation study, we extend the work of Ren et al. [19] where a variety of causal tools were evaluated to estimate treatment effects with both measured and unmeasured confounders.

Ren et al. compare G-computation, Inverse Probability of Treatment Weighting (IPTW) and TMLE approaches. The methods which performed best, [G-computation (GC) and IPTW using Overlapping Weighting (PS_OW)] are retained here and compared against the Model Estimated Controls (MEC) method proposed here. It should be noted that neither the GC or PS_OW approaches are feasible in the intended setting for MEC but are included here as a comparison. A direct comparison of treatments without any correction for potential confounding is also performed (RAW). Following the notation and conditions of Ren et al. define baselined covariates (C), treatment allocations (A), measured confounders (L), unmeasured confounders (U) and patient outcomes (Y). Data are simulated under three scenarios;

1. Weak connections between unmeasured confounders (U) and measured confounders (L) but with no direct relationship between U and A.
2. Moderate connections between unmeasured confounders (U) and measured confounders (L) but with no direct relationship between U and A.
3. Moderate connections between unmeasured confounders (U) and measured confounders (L) and with a direct relationship between U and A.

We apply the same rules for simulating datasets for the simulation study whilst setting a different random seed. To ensure the simulation study is representative of the intended setting, we also ensure that the number of patients who receive the control treatment and who a counter factual model is generated outweigh those who received the experimental treatment.

Initially a dataset of 30,000 patients is simulated. For each dataset, outcome data are sampled to represent continuous data, binary data and survival data. For each simulation 600 patients are sampled, 500 patients who received the control treatment and 100 patients who received the experimental treatment. To the 500 patients who received the control treatment models are fit to estimate the outcome based on both the baseline covariates (C) and measured confounders (L). For continuous outcome, a GLM with an identity link and Gaussian distribution is used, for binary outcome a logit link and a binomial family is used and for survival data a flexible parametric model with 3 internal knots is used. The likelihood estimation procedure detailed in Sect. 2 is used to estimate the $\:\beta\:$ parameter which is used to measure the difference between experimental and control treatments. Code is provided for the simulation study as part of the supplementary material.

**Fig. 1:**
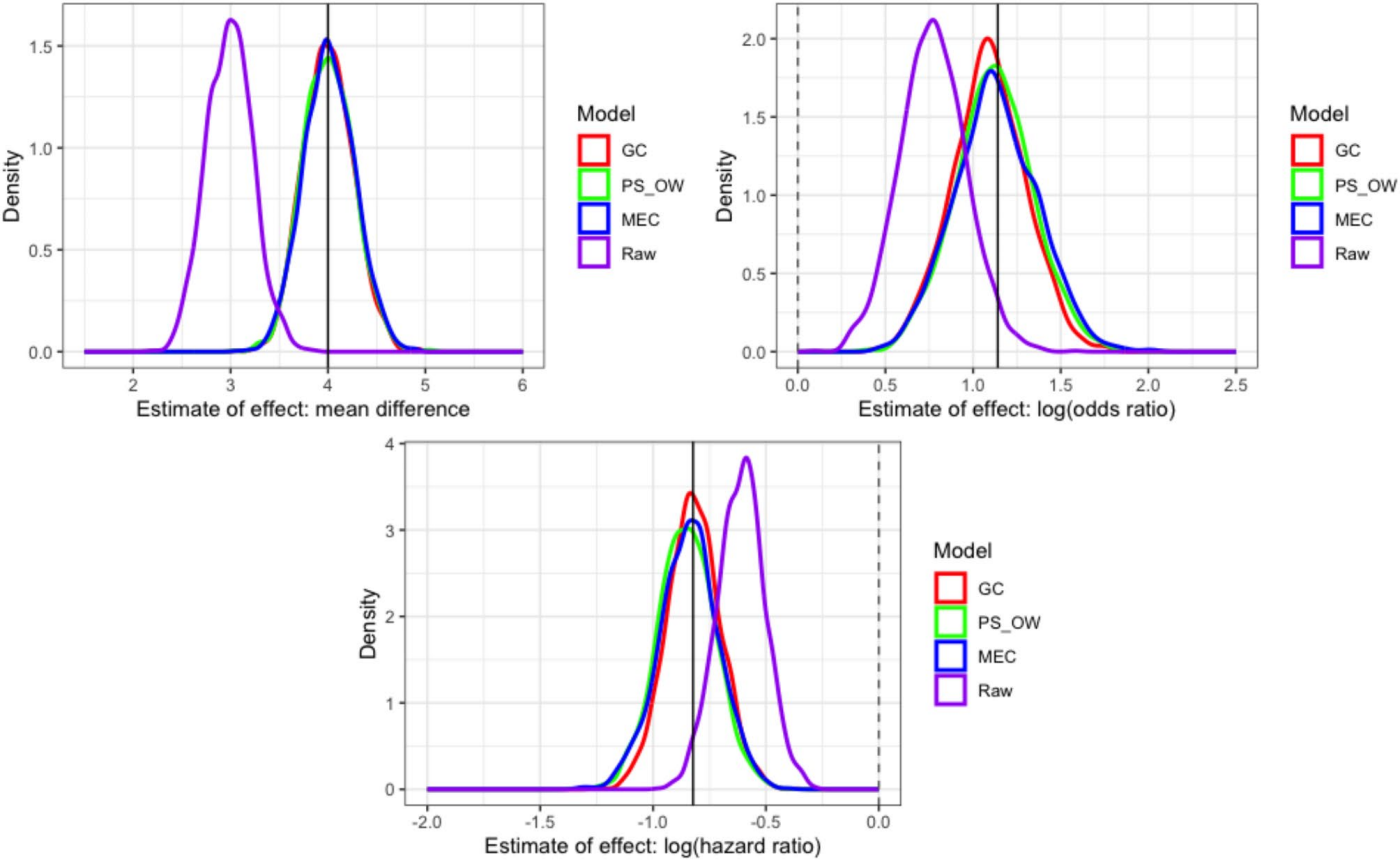
Density plots of bias for simulation scenario 2 (moderate known confounding)

Table 2 gives the comparative results of each method to each scenario. The fitted results of scenario 2 are given in Fig. 1. Figures for the results of Scenarios 1 and 3 are provided in the supplementary material. The results show that in terms of bias, there is comparative performance of the three methods applied in all scenarios. For Scenario 1 and 2 there is evidence that the coverage for the GC method is preferential for continuous outcomes. For a binary outcome, coverage for GC and MEC outperform PS_OW. For scenario 3 with unmeasured confounding, none of the methods completely account for bias as may be expected although there is some evidence of MEC outperforming GC and PS_OW in terms of bias and coverage for binary and survival outcomes.

**Table 2:** Results of a simulation study estimating the efficacy between treatments ($\:\beta\:)$ for G-Computation (GC), propensity scores with overlapping weights (PS_OW), model estimated controls (MEC) and unadjusted comparisons (Raw) in 3 scenarios with differing assumptions of confounding

		Model	bias	var	coverage	width
Scenario 1	Continuous	GC	**-0.00522**	0.04678	0.9485	0.85604
		MEC	**-0.00255**	0.04915	0.897	0.72372
		PS_OW	**-0.00597**	0.04896	0.861	0.65271
		Raw	**-0.78427**	0.03776	0.0225	0.78853
	Binary	GC	**0.06485**	0.05019	0.9435	0.89167
		MEC	**0.1022**	0.06003	0.9455	1.02261
		PS_OW	**0.06583**	0.05414	0.8355	0.65905
		Raw	**-0.18126**	0.0407	0.837	0.79239
	Survival	GC	**-0.0046**	0.01396	0.9535	0.46877
		MEC	**-0.02067**	0.01768	0.9415	0.5092
		PS_OW	**-0.027**	0.01613	0.9435	0.50483
		Raw	**0.17439**	0.01159	0.6185	0.4246
Scenario 2	Continuous	GC	**0.00207**	0.06516	0.952	1.00352
		PSC	**0.01391**	0.06939	0.865	0.79171
		PS_OW	**0.00562**	0.06871	0.8655	0.78167
		Raw	**-1.00956**	0.05667	0.015	0.93492
	Binary	GC	**-0.0499**	0.04489	0.9455	0.8496
		MEC	**-0.01015**	0.05636	0.969	1.01531
		PS_OW	**-0.02036**	0.04947	0.847	0.64569
		Raw	**-0.3694**	0.03597	0.5165	0.76179
	Survival	GC	**0.00279**	0.01383	0.9455	0.4594
		MEC	**-0.0192**	0.01736	0.9415	0.50773
		PS_OW	**-0.02799**	0.01618	0.942	0.49698
		Raw	**0.21236**	0.01119	0.474	0.42193
Scenario 3	Continuous	GC	**-0.47753**	0.06561	0.537	0.99825
		MEC	**-0.48421**	0.0709	0.3745	0.79975
		PS_OW	**-0.49079**	0.06893	0.3425	0.76354
		Raw	**-1.4137**	0.05514	0	0.91146
	Binary	GC	**-0.21875**	0.04899	0.822	0.86272
		MEC	**-0.19293**	0.0592	0.894	1.01731
		PS_OW	**-0.21718**	0.05375	0.655	0.64003
		Raw	**-0.50107**	0.03861	0.2665	0.75876
	Survival	GC	**0.05859**	0.01342	0.923	0.46567
		MEC	**0.03043**	0.01784	0.9385	0.5143
		PS_OW	**0.04353**	0.01505	0.948	0.49976
		Raw	**0.24478**	0.01101	0.372	0.42127

## Example

The methodology is applied to the survival data from two trials of the European Study group for Pancreatic Cancer (ESPAC). The ESPAC-3 study [20] randomized 1088 patients to receive either Gemcitabine (Gem) or 5-Fluorouracil (5FU) as adjuvant therapies for patients with Pancreatic Ductal Adenocarcinoma (PDAC). Whilst the study failed to show any differences in overall survival, Gem demonstrated a superior toxicity profile and became the standard of care. Following this, the ESPAC-4 [21] study recruited 732 patients to receive either Gem or a combination therapy, GemCap.

The aim here is to use the data from the initial trial (ESPAC3) to generate a CFM for patients receiving Gem. This model will then be used as a control against which the patients receiving GemCap in ESPAC4 will be compared. Alongside this a direct comparison of the two treatments from ESPAC4 will be made. This allows for an empirical comparison of evidence obtained from the model generated controls against that obtained from an RCT. A schematic of this approach is included in Supplementary Sect. 1.1.

### CFM model (Gemcitabine)

Given that reliable estimation of a treatment effect depends on a reliable CFM, some attention is given to the development and validation of the parametric model for patients receiving Gem. The multivariable parametric model used is constructed using a flexible spline model [15] including a single internal node for the estimation of the baseline hazard function. Validation of this model is carried out with the Gem patients from ESPAC-4 using measures of fit, discrimination and calibration [22]. Details on the validation of this model are included in Supplementary Sect. 1.2.

### Estimation

Estimation is performed using the approach detailed in Supplementary Sect. 1.3 using the ‘psc’ package developed within R for this purpose available from https://www.github.com/richjjackson/psc which is coded in R (Version 4) [23]. This is based on the conditional likelihood for survival outcomes. Prior distributions are defined based on hyperparameters taken directly from the CFM and a vague uninformative prior for the log hazard ratio is defined as $\:\pi\:\left(\beta\:\right)\sim\:N\left(\text{0,1000}\right)$. Posterior distributions are generated from a routine which includes 25,000 iterations from 3 chains and inclusive of a 5000 burn in and applying a thin of 10 [16].

### Results

**Fig. 2:**
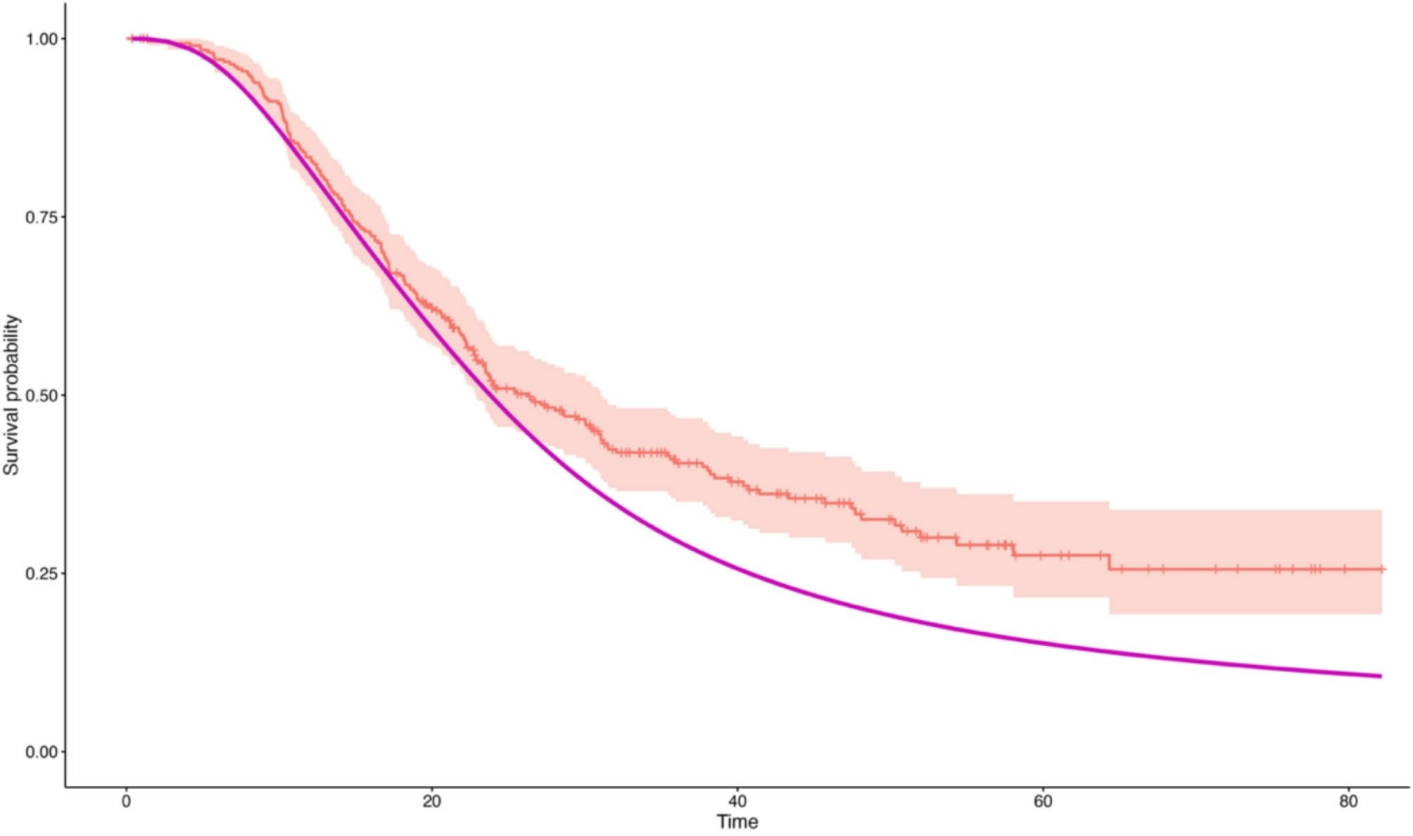
Comparison of model estimated control for Gem (purple) vs. observed survival outcome for GemCap (red)

Graphical representations of the model fit are included in Supplementary Fig. 3 and show histograms of the prognostic index for both the Gem and the GemCap populations from the ESPAC-4 dataset. The trace obtained for the log hazard ratio is included to show stable convergence has been obtained within the MCMC procedure and the resulting posterior density is given alongside the observed median and 2.5% and 97.5% quantiles. Figure 2 shows the observed survival estimates obtained from the data cohort (GemCap) along with the model estimated control being the expected survival of the equivalent population had they received the control treatment (Gem). This is obtained by multiplying the cumulative baseline hazard function by the mean of the linear predictor.

Table 3 shows the estimate of the hazard ratio of GemCap versus Gem and 95% credibility interval obtained as the highest posterior density. Also shown is the hazard ratio obtained by evaluating GemCap patients from ESPAC-4 against the Gem patients from ESPAC-4. The results show that comparing the GemCap cohort against the CFM gives an estimates hazard ratio of 0.78 (0.59–1.03) compared to a hazard ratio of 0.79 (0.65–0.96) when GemCap is evaluated against to Gem directly using only the ESPAC-4 data. It should be noted that there is good agreement in the point estimate of the hazard ratio. The standard error obtained is greater for PSC approach when compared to that obtained via direct estimation (0.14 vs. 0.10). This may be because the ‘Gem’ population using the model generated controls approach is based on fewer patients (*n* = 191) than that using direct estimation (*n* = 220). It should also be noted however that the model estimated controls approach does produce estimates with a lower degree of precision compared to estimating efficacy of two randomised populations.

**Table 3:** Estimation of hazard ratio for the comparison of GemCap vs Gem

			Model Estimated Controls			Direct Estimation	
			$\:\widehat{\beta\:}$(se)	Hazard Ratio (95% CI)		$\:\widehat{\beta\:}$(se)	Hazard Ratio (95% CI)
Full Population			-0.250 (0.14)	0.78 (0.59–1.03)		-0.239 (0.10)	0.79 (0.65–0.96)
Sub-Group Analyses	Lymph Nodes	Negative	-0.33 (0.19)	0.72 (0.47–1.05)		-0.28 (0.14)	0.75 (0.572, 0.99)
		Positive	-0.15 (0.17)	0.86 (0.61–1.16)		-0.19 (0.14)	0.82 (0.62, 1.092)
	T Stage	2	-0.48 (0.48)	0.62 (0.26–1.12)		-1.00 (0.48)	0.37 (0.144, 0.936)
		3	-0.13 (0.19)	0.88 (0.61–1.27)		-0.18 (0.12)	0.84 (0.655, 1.066)
		4	-0.33 (0.25)	0.72 (0.43–1.14)		-0.21 (0.19)	0.81 (0.561, 1.163)
	Tumour Grade	Moderate	-0.23 (0.18)	0.79 (0.54–1.10)		-0.14 (0.15)	0.87 (0.655, 1.162)
		Poor	-0.24 (0.19)	0.79 (0.51–1.18)		-0.34 (0.15)	0.71 (0.537, 0.947)
	CA19.9	< 20	-0.37 (0.17)	0.69 (0.47–0.93)		-0.27 (0.15)	0.76 (0.57, 1.02)
		≥ 20	-0.15 (0.16)	0.86 (0.59–1.15)		-0.13 (0.13)	0.87 (0.69, 1.11)

### Sub-group analyses

The results in Table 2 extend the analysis to explore possible subgroup effects, which are then compared with equivalent results obtained via the direct comparison of GemCap against Gem in the ESPAC-4 study. Subgroup analyses using model estimated controls are performed by restricting the population over which the comparison is made. Inspection of Table 3 shows a good level of agreement between the subgroup effects obtained using both estimation approaches. There is loss of precision, which is to be expected as patient numbers reduce, but it is observed that all point estimates for the CFM are within one standard error of the equivalent estimates obtained via direct estimation.

The purpose here was to demonstrate that the model estimated controls can reliably be applied and offer an alternative to direct randomisation of prospective patients. In this instance the point estimate of the comparison between the Gem and GemCap are comparable using the two approaches and this suggests that future studies could theoretically have been performed using model estimated controls to replace prospectively recruited control patients.

## Discussion

Here we explore the possibility of estimating a treatment effect by comparing a cohort of data against counter-factual evidence obtained from a parametric model. We generate two approached based on firstly simulating data from a model and secondly, a likelihood approach which can handle several outcomes. We propose a Bayesian procedure to provide reliable estimation. Code to perform this estimation has been developed into an R package (github.com/richjjackson/psc) with an example for their general application included in Supplementary Sect. 3.

A simulation study has been performed which demonstrates that the performance of model estimated controls is comparable to G-computation and Propensity score estimation in presence of known and unknown confounders in terms of bias and coverage.

As this approach utilises model predictions there are some similarities with G-computational approaches, aside from differences in setting, there is also a difference in estimation as G-computation compares expected outcomes from each treatment group whereas using model estimated controls compares an observed outcome (from the data) against an expected outcome (from the model). Parallels may also be drawn to the missing data problem posed by Imbens and Rubins [17]. In this context, the counter factual model may be thought of as an imputation tool. The inclusion of a likelihood to directly estimate the efficacy parameter and an estimation procedure to estimate precision also separates out model estimated controls from these approaches.

Practically, model estimated controls are useful in a different setting to other causal inference tools which are applied where data are available on both treatment arms being compared. Here it is required that data only on the experimental arm are available and applications are envisaged in the use of registry databases, the design of prospective clinical trials, where models could be used to replace, in part or in full, prospectively recruited patients and the evaluation of completed RCTs.

We have demonstrated that MECs can work well with an application to two RCTs in Pancreatic Cancer where the first RCT is used to develop a CFM and the second supplies the data cohort. It should be noted that it may be expected that the method performs well in this setting as both the CFM and the data cohort were taken from randomised trials in comparable patient populations in studies administered by the same trials unit. Both sources of information then had the protection of randomisation and there was little concern on model extrapolation or transportability. Further review of the use of this MECs in observational settings is planned as part of the future development.

Strengths of the proposed approach are the new setting in which they are intended and the comparable performance to other leading causal inference tools. We have also ensured the method is accessible by producing a package within R for its application. As with other tools however, its use is dependent on a set of assumptions which may not be testable. Most notably the assumption of no unmeasured confounders which have the potential to bias any comparison and the assumption that it is reasonable to transport the model from the setting in which it was developed.

The basis for this method is also a good model on which to generate the counter factual evidence. There is the inherent assumption that model is appropriate for the setting in which it is being used, and care should be applied to ensure that models are not being extrapolated beyond their intended use. There is some assurance available however as models can be validated to ensure they provide accurate predictions. Where validation is possible, this is likely due to data being available on both experimental and control arms and here some consideration should be given as to how model estimated controls may be used in conjunction with other available methodologies (e.g. G-estimation or TMLE).

In summary, there is a benefit and opportunity to use methodology which used predictive models to generate counter-factual evidence, and this can be a form of causal inference which is shown to be reliable and comparable to other causal tools.

There are also further developments and applications beyond what have been explored here. Using model generated controls could be used as a calibration tool for model validation (where the model and data represent the same treatment). It is also being explored as a tool to predict future phase III outcomes from a single arm phase II study using posterior predictive distributions.

Two estimation procedures are presented, one which attempts to directly simulate control data and a second which develops a likelihood approach. In its current form only estimating an ATE has been explored, either directly or through use of a linear term using the likelihood approach. There are opportunities to develop both approaches to explore alternative relationships (e.g. multiplicative effects, interaction effects or non-proportional hazards). Further extensions include allowing for random effects (including longitudinal models) and developing estimation procedures for non-parametric/semi-parametric model alternatives to the CFM, including those obtained from machine learning tools, as well as the potential to explore doubly robust estimators similar to those applied in TMLEs.

There are also subtle differences in the causal estimand obtained using model estimated controls to most other forms of causal estimation. Whereas most methods estimate a treatment effect at a population level, requiring assumptions of balance in confounders to exist between treated and non-treated groups at a population level. Model estimated controls work by estimating the counter factual evidence at a patient level. In so doing they can theoretically estimate efficacy where there is not balance between treated and untreated populations. This also may make them more suitable to investigate measurement of treatment effect heterogeneity and the estimation of personalised treatment effects [23, 24].

## Electronic supplementary material

Below is the link to the electronic supplementary material.

Supplementary Material 1

## Data Availability

The example of R codes for data simulation and analysis can be found in the supplementary file and further details on the application of the methodology are available at gihub.com/richjjackson/psc. Data to replicate the analysis of trial data are available from the author upon request.
