# Supplementary material for: Estimating treatment effects using parametric models as counter-factual evidence
Source: BMC Med Res Methodol. 2025 Apr 9;25:91. doi: 10.1186/s12874-025-02540-2 (PMC11983918; doi:10.1186/s12874-025-02540-2)
Supplement: Supplementary file 1 — Supplementary Material 1 [file 12874_2025_2540_MOESM1_ESM.docx]

# Supplementary Material

## Section 1: Details on the comparison of GemCap Vs Gem

### Section 1.1 Analysis Schematic

Details are provided on the process of using Model Estimated Controls to derive a ‘CFM’ from ESPAC3 (Gemcitabine) for the comparisons of the experimental treatment in ESPAC-4 (Gemcitabine + Capecitabine). Supplementary Figure 1 provides a schematic of this comparison. Further details are available on a github repostirory which acts as a companion to this manuscript found at richjjackson.github.com/modelEstimatedControls

#### Supplementary Figure 1: Schematic on the use of ESPAC-3 and ESPAC-4 data

*
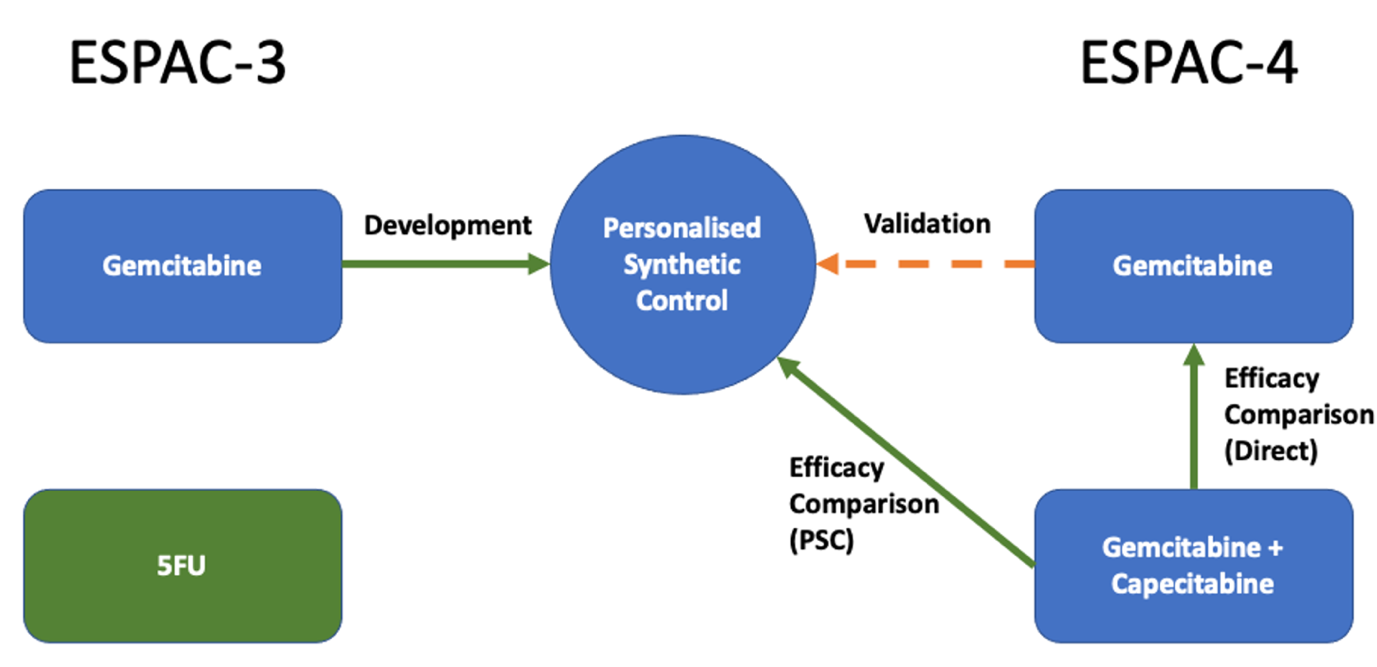
*

### Section 1.2 Validation of model to predict survival under Gemcitabine

Validation of a CFM to estimate the survival of patients receiving Gemcitbine (Gem) is carried out with the patients from ESPAC-4 using measures of fit, discrimination and calibration [13].

First the prognostic index (PI) is used to create four risk groups based on the 15^th^, 50^th^ and 85^th^ percentiles of the PI values. Equivalent risk groups are obtained for the Gem patients in both the ESPAC-3 and ESPAC-4 datasets. For each risk group, an estimate of the survival distribution is obtained using the mean of the survival function for each observation in each risk group. This allows comparisons between observed Kaplan Meier survival estimates and predicted survival estimates from the fitted model. Model fit is demonstrated visually in Supplementary Figure 3. Figure 3a shows this comparison for patients from ESPAC-3 on whom the model was trained. Figure 3b shows the model estimates against observed survival estimates for patients in ESPAC-4. Visually there is a good level of agreement between the two estimates and good levels of discrimination between each risk group.

Supplementary Table 1 gives further details on model validation, comparing the median (95% CI) overall survival estimates within each risk group and hazard ratios (95% CI) comparing survival between the risk groups in both the ESPAC-3 and ESPAC-4 populations. Further validation measures include the regression slope where the PI is regressed against patient outcome, with values significantly different from 1 representing under/over discrimination. Concordance is measured using Harrell’s concordance index [14] and measures of explained variation using Royston’s (2006) D [15].

#### Supplementary Table 1. Results of validation of parametric model for patients receiving gemcitabine for patients receiving gemcitabine

|  |  | Derivation Model (ESPAC-3) | Validation Model (ESPAC-4) |
| --- | --- | --- | --- |
| Median OS (95% CI) | Group 1 (Low Risk) | 43.4 (31.7, 97.4) [n=54] | 59.8 (41.03 - Undefined) [n=46] |
|  | Group 2 (Fairly Low Risk) | 27.6 (24.9, 33.6) [n=126] | 32.8 (26.25, 49.0) [n=85] |
|  | Group 3 (Fairly High Risk) | 20.1 (18.5, 23.0) [n=125] | 16.7 (13.99, 22.10) [n=55] |
|  | Group 4 (High Risk) | 12.8 (10.3, 16.8) [n=54] | 12.3 (8.11, 22.6) [n=34] |
| Haz Ratio (95% CI) | 1 Vs 2 | 1.73 (1.18, 2.54) | 1.70 (0.98, 2.96) |
|  | 1 Vs 3 | 2.79 (1.90,4.10) | 4.01 (2.31, 6.98) |
|  | 1 VS 4 | 5.23 (3.37, 8.13) | 5.34 (2.95, 9.66) |
| Discrimination Measures | Regression Slope | - | 1.18 (0.168) |
|  | Harell’s C | 0.637 (0.018) | 0.682 (0.025) |
|  | Royston's D | 0.137 (0.102) | - 1. (0.217) |

#### Supplementary Figure 2. Visualisation of the fitted survival function for Gem from ESPAC-3 and the unadjusted Kaplan Meier estimates of survival from ESPAC-4. Figures show fitted comparisons of the survival for Gem within the 4 risk groups derived from the prognostic index (PI) for a) ESPAC3 and b) ESPAC4 and c) comparison of the observed Kaplan Meier estimates for GemCap against the model averaged effects of Gem obtained from the PSC.

**

### Section 1.3 Estimation of Treatment effect comparing GemCap Vs Gem in Pancreatic Ductal Adenocarcinoma

#### Hyperparameters for the definition of prior distributions

The prior distribution containing the information for the CFM in are defined based on

$$\pi(B)\sim MVN(\mu,\Sigma)$$

Where

$$\mu= \left[ -10.08, 2.88, 0.27, 0.23, 0.20, 0.20, 0.29, 0.52, 0.15, 0.47 \right]$$

and

$$\Sigma= \left[ \begin{matrix} \begin{matrix} \begin{matrix} 0.392 & -0.116 \\ -0.116 & 0.042 \end{matrix} & \begin{matrix} -0.015 & -0.006 \\ 0.006 & 0.001 \end{matrix} & \begin{matrix} -0.004 & -0.014 \\ -0.001 & 0.000 \end{matrix} \\ \begin{matrix} -0.015 & 0.006 \\ -0.006 & 0.001 \end{matrix} & \begin{matrix} 0.001 & 0.000 \\ 0.000 & 0.001 \end{matrix} & \begin{matrix} 0.000 & 0.000 \\ -0.001 & 0.000 \end{matrix} \\ \begin{matrix} 0.004 & -0.001 \\ -0.014 & 0.000 \end{matrix} & \begin{matrix} 0.000 & -0.001 \\ 0.000 & 0.000 \end{matrix} & \begin{matrix} 0.014 & 0.000 \\ 0.000 & 0.016 \end{matrix} \end{matrix} & \begin{matrix} \begin{matrix} -0.010 & -0.013 \\ 0.001 & 0.000 \end{matrix} & \begin{matrix} -0.036 & -0.036 \\ 0.001 & 0.002 \end{matrix} \\ \begin{matrix} 0.000 & 0.000 \\ -0.001 & -0.001 \end{matrix} & \begin{matrix} 0.000 & 0.000 \\ 0.001 & 0.000 \end{matrix} \\ \begin{matrix} 0.001 & 0.000 \\ 0.010 & 0.000 \end{matrix} & \begin{matrix} 0.002 & 0.002 \\ 0.002 & 0.001 \end{matrix} \end{matrix} \\ \begin{matrix} \begin{matrix} -0.010 & 0.001 \\ -0.013 & 0.00 \end{matrix} & \begin{matrix} 0.000 & -0.001 \\ 0.000 & -0.001 \end{matrix} & \begin{matrix} 0.001 & 0.010 \\ 0.000 & 0.000 \end{matrix} \\ \begin{matrix} -0.036 & 0.001 \\ -0.036 & 0.002 \end{matrix} & \begin{matrix} 0.000 & 0.001 \\ 0.000 & 0.000 \end{matrix} & \begin{matrix} 0.002 & 0.002 \\ 0.002 & 0.001 \end{matrix} \end{matrix} & \begin{matrix} \begin{matrix} 0.041 & 0.000 \\ 0.000 & 0.020 \end{matrix} & \begin{matrix} 0.000 & -0.002 \\ -0.001 & -0.002 \end{matrix} \\ \begin{matrix} 0.000 & -0.001 \\ -0.002 & -0.002 \end{matrix} & \begin{matrix} 0.034 & 0.029 \\ 0.029 & 0.041 \end{matrix} \end{matrix} \end{matrix} \right]$$

Here the first 3 components of $\mu$ relate to estimation of the cumulative baseline hazard function and the remaining components relate to the parameters associated with estimating the linear predictor

#### Results of the Bayesian estimation procedure

Results of the model fitting procedure including a) PI obtained from the ESPAC-3 data, b) PI obtained from the ESPAC-4 data, c) trace of the posterior distribution for the hazard ratio (β) and d) the posterior density of hazard ratio (β) with median and 95% highest posterior density.

#### Supplementary Figure 3: Results of Bayesian estimation

**

### Section 1.4 Comparison of likelihood and non-parametric estimation of efficacy parameters

*Comparison of the the efficacy parameter as obtained from two different estimation procedures are presented. Here models for binary, continuous and survival endpoints are selected along with an example dataset available as part of the psc package in R. To each model, both estimation procedures are applied and the resulting efficacy parameters are compared.*

#### Section 1.5 MCMC Sampling routine to obtain marginal estimate of the efficacy parameter

For each iteration of the MCMC procedure, the following algorithm is performed

1. Set and indicator s=1
2. Update s = s+1 and draw model parameters $b_{s}$ from $\pi\left( B \right)$ and a draw a proposal estimates $\tau_{s}$ from some target distribution for $\beta$
3. Estimate $\gamma_{i,S}=b^{T}x_{i}$ where $x$contains the data covariates and define
   1. $\vartheta_{s,1}=L\left( D | B=b_{s},\beta=\tau_{s-1} \right)$
   2. $\vartheta_{s,2}= L(D|B=b_{s},\beta=\tau_{s})$
4. Draw a single value $\varsigma$ from a Uniform (0,1) distribution and estimate the condition $\omega= \frac{\vartheta_{s,2}}{\vartheta_{s,2}}$
   1. If $\omega$ > $\varsigma$ then accept $\tau_{s}$ as belonging to the marginal posterior distribution $P(\beta)$ otherwise retain $\tau_{s-1}$
5. Repeat steps 2 – 4 for the required number of iterations

The result of the algorithm is a posterior distribution for the marginal distribution of $\beta$ [$P(\beta|D)$] which captures the variability in B through $\pi\left( B \right)$.

## Section 2: R code used to perform simulation study

### Simulation study - comparing with G estimation and PS

library(psc)

library(boot)

library(simstudy)

library(RISCA)

library(survival)

library(tidyr)

library(data.table)

library(flexsurv)

library(plotly)

library(simhelpers)

library(knitr)

library(gridExtra)

## Sim Data

### Registry Data

### Single Arm Clinical Trial

### Function for estimating PS weights

getWeight <- function(predA0, actA0, method) {

if(method==1){

wt <- 1/ (actA0*predA0 + (1-actA0)*(1-predA0)) # IPTW

}else if(method==2){

wt <- actA0*(1-predA0) + (1-actA0)*predA0 }else if(method==3){

wt <- actA0*1 + (1-actA0)*predA0/(1-predA0) # SMR

# Overlapping weighting

}

return(wt)

}

###### Data Simulaton (obtained from Ren et al.)

##################################################################################

# G-computation versus propensity score-based methods

#

# Establish a comparable control arm for a single-arm clinical trial

# (Cope with baseline covariates only)

#--------------------------------------------------------------------------------

#Data set

# C: fixed baseline covariate, e.g. age

# L0: time-varying covariate at baseline, e.g. ECOG performance status 0-1 vs 2+

# A0: time-varying covariate at baseline, e.g. treatment

# Ya: continuous outcome, e.g. mobility score

# Yb: binary outcome, e.g. treatment response

# Yc: time-to-event outcome, e.g. overall survival

# D: randomly censoring outcome, e.g. 1-Death

##################################################################################

#--------------------------------------------------

#Step 1: Create a data set (with treatment effect)

#consider as a target population

#--------------------------------------------------

set.seed(041084)

defLSM<- defData(varname = "U", formula = 0.5, variance = 1, dist = "normal")

defLSM<- defData(defLSM, varname = "C", formula = 50, variance = 50, dist = "normal")

defLSM<- defData(defLSM, varname = "e0", formula = 0, variance = 2, dist = "normal")

defLSM<- defData(defLSM, varname = "L0", formula = "-2.66+ U + e0",

dist = "binary", link = "logit")

defLSM<- defData(defLSM, varname = "e1", formula = 0, variance = 1, dist = "normal")

defLSM<- defData(defLSM, varname = "A0", formula = "-13.42 + L0*1.5 + C*0.25 + e1",

dist = "binary", link = "logit")

defLSM<- defData(defLSM, varname = "e2", formula = 0, variance = 4, dist = "normal")

defLSM<- defData(defLSM, varname = "Ya",

formula = "50 - C*0.1 - U*0.5 + A0*4 + e2",

dist = "nonrandom")

defLSM<- defData(defLSM, varname = "e3", formula = 0, variance = 4, dist = "normal")

defLSM<- defData(defLSM, varname = "Yb",

formula = "2.5 - C*0.05 - U*0.5 + A0*2 + e3",

dist = "binary", link = "logit")

defLSM<- defData(defLSM, varname = "X", formula = "0;1", dist = "uniform")

defLSM<- defData(defLSM, varname = "e4", formula = 0, variance = 0.4, dist = "normal")

defLSM<- defData(defLSM, varname = "Yc",

formula = "(-log(X)*exp(5.0 - C*0.03 - U*0.15 - A0*log(1/3) + e4))^(1/1.2)",

dist = "nonrandom")

defLSM<- defData(defLSM, varname = "D",

formula = 0.8,

dist = "binary")

set.seed(41084)

dt <- genData(30000, defLSM)

#---------------------------------

#Step 2: set up the true effect

#---------------------------------

# Mean difference

fit_ya <- glm(Ya ~ A0 + L0 + U + C, data=dt)

true_ya = matrix(fit_ya$coefficients)[2,1]

# Odds Ratio

fit_yb<- glm(Yb ~ A0 + L0 + U + C, family=quasibinomial("logit"), data=dt)

gc.yb<- gc.logistic(glm.obj=fit_yb, data=dt, group="A0", effect="ATE",

var.method='bootstrap', iterations=2, n.cluster=1)

true_yb = gc.yb$logOR[,1]

# Hazard ratio

fit_yc<- coxph(Surv(Yc, D) ~ A0 + U + C, data=dt)

gc.yc<- gc.survival(object=fit_yc, data=dt, group="A0", time='Yc', failure='D', effect="ATE",

max.time=max(dt$Yc), iterations=2, n.cluster=1)

true_yc = gc.yc$logHR[,1]

### Set working directory for output

setwd("~/Documents/GitHub/pscSim/output")

#---------------------------------

#Step 3: sampling and calculation

#---------------------------------

#Initial value

n_sim = 2000

n_patients_0 = 500

n_patients_1 = 150

n_methods = 4

### Setting Scenario

dt <- dtL

output_ya = data.frame(matrix(data=NA, n_sim*n_methods, 4))

output_yb = data.frame(matrix(data=NA, n_sim*n_methods, 4))

output_yc = data.frame(matrix(data=NA, n_sim*n_methods, 4))

colnames(output_ya)<- c("Model", "A0_est", "A0_lower", "A0_upper")

colnames(output_yb)<- c("Model", "A0_est", "A0_lower", "A0_upper")

colnames(output_yc)<- c("Model", "A0_est", "A0_lower", "A0_upper")

dt0 <- dt[dt$A==0,]

dt1 <- dt[dt$A==1,]

for (i in 1:n_sim){

print(i)

sim0 <- dt0[dt0$id %in% sample(dt0$id, n_patients_0, replace = F),]

sim1 <- dt1[dt1$id %in% sample(dt1$id, n_patients_1, replace = F),]

sim <- rbind(sim0,sim1)

#sim = dt[dt$id %in% sample(dt$id, n_patients, replace = F),]

#Method 1: Propensity score-based

# function to convert propensity scores to IPW

fitA0 <- glm(A0 ~ L0 + C, data = sim, family=binomial)

sim[, predA0 := predict(fitA0, type = "response")]

sim[, ow:= getWeight(predA0, A0, 2)]

sim=data.table(sim)

#PS-OW

fit_ya = glm(Ya ~ A0 , weights = ow, data = sim)

output_ya[1+n_methods*(i-1), 1] = "PS_OW"

output_ya[1+n_methods*(i-1), 2] = fit_ya$coefficients[2]

output_ya[1+n_methods*(i-1), 3:4] = confint.default(fit_ya)[2, 1:2]

fit_yb = glm(Yb ~ A0 , family=quasibinomial("logit"), weights = ow, data = sim)

output_yb[1+n_methods*(i-1), 1] = "PS_OW"

output_yb[1+n_methods*(i-1), 2] = fit_yb$coefficients[2]

output_yb[1+n_methods*(i-1), 3:4] = confint.default(fit_yb)[2, 1:2]

fit_yc = coxph(Surv(Yc, D) ~ A0, weights = ow, data = sim)

output_yc[1+n_methods*(i-1), 1] = "PS_OW"

output_yc[1+n_methods*(i-1), 2] = fit_yc$coefficients[1]

output_yc[1+n_methods*(i-1), 3:4] = confint.default(fit_yc)[1, 1:2]

#Method 2: G-computation

#Bootstrap (re-sampling) for Ya

ya_boots = matrix(NA, 100, 1)

for (j in 1:100) {

id <- sort(sample(unique(sim$id), 1*length(unique(sim$id)), replace=T)) #Bootstrap (with replacement)

id <- data.frame(cbind(id, IDNEW=c(1:length(id))))

sim_ya<- merge(sim, id, by="id")

fit_ya<- glm(Ya ~ A0 + L0 + C, data = sim_ya)

sim1 = sim_ya

sim1$A0 = 1

sim0 = sim_ya

sim0$A0 = 0

ya_boots[j, 1] = mean(predict(fit_ya, sim1)) - mean(predict(fit_ya, sim0))

}

output_ya[2+n_methods*(i-1), 1] = "GC"

output_ya[2+n_methods*(i-1), 2] = mean(ya_boots)

output_ya[2+n_methods*(i-1), 3] = mean(ya_boots) - qnorm(0.975)*sqrt(cov(ya_boots))

output_ya[2+n_methods*(i-1), 4] = mean(ya_boots) + qnorm(0.975)*sqrt(cov(ya_boots))

fit_yb<- glm(Yb ~ A0 + L0 + C, family=quasibinomial("logit"), data = sim)

gc.yb<- gc.logistic(glm.obj=fit_yb, data = sim, group="A0", effect="ATE",

var.method='bootstrap', iterations=100, n.cluster=1)

output_yb[2+n_methods*(i-1), 1] = "GC"

output_yb[2+n_methods*(i-1), 2] = gc.yb$logOR[,1]

output_yb[2+n_methods*(i-1), 3:4] = gc.yb$logOR[,3:4]

fit_yc<- coxph(Surv(Yc, D) ~ A0 + L0 + C, data = sim)

gc.yc<- gc.survival(object=fit_yc, data = sim, group="A0", time='Yc', failure='D', effect="ATE",

max.time=max(sim$Yc), iterations=100, n.cluster=1)

output_yc[2+n_methods*(i-1), 1] = "GC"

output_yc[2+n_methods*(i-1), 2] = gc.yc$logHR[,1]

output_yc[2+n_methods*(i-1), 3:4] = gc.yc$logHR[,3:4]

#Method 3: PSC

sim.control <- sim[sim$A0==0,]

sim.exp <- sim[sim$A0==1,]

cont_mod<- glm(Ya ~ L0 + C, data = sim.control)

cont.psc <- pscfit(cont_mod,sim.exp)

output_ya[3+n_methods*(i-1), 1] = "PSC"

output_ya[3+n_methods*(i-1), c(2,3,4)] <- coef(cont.psc)[1,c(1,2,3)]

bin_mod<- glm(Yb ~ L0 + C,family="binomial", data = sim.control)

bin.psc <- pscfit(bin_mod,sim.exp)

output_yb[3+n_methods*(i-1), 1] = "PSC"

output_yb[3+n_methods*(i-1), c(2,3,4)] <- coef(bin.psc)[1,c(1,2,3)]

s.ob <- Surv(sim.control$Yc,sim.control$D)

sim.control[1:3,]

surv_mod<- flexsurvspline(s.ob ~ L0 + C,k=3, data = sim.control)

sim.exp$cen <- sim.exp$D

sim.exp$time <- sim.exp$Yc

options(warn=-1)

surv.psc <- pscfit(surv_mod,sim.exp)

output_yc[3+n_methods*(i-1), 1] = "PSC"

output_yc[3+n_methods*(i-1), c(2,3,4)] <- coef(surv.psc)[1,c(1,2,3)]

#Method 4: Unadjusted (raw)

fit_ya = glm(Ya ~ A0, data = sim)

output_ya[4+n_methods*(i-1), 1] = "Raw"

output_ya[4+n_methods*(i-1), 2] = fit_ya$coefficients[2]

output_ya[4+n_methods*(i-1), 3:4] = confint.default(fit_ya)[2, 1:2]

fit_yb = glm(Yb ~ A0 , family=quasibinomial("logit"), data = sim)

output_yb[4+n_methods*(i-1), 1] = "Raw"

output_yb[4+n_methods*(i-1), 2] = fit_yb$coefficients[2]

output_yb[4+n_methods*(i-1), 3:4] = confint.default(fit_yb)[2, 1:2]

fit_yc = coxph(Surv(Yc, D) ~ A0, data = sim)

output_yc[4+n_methods*(i-1), 1] = "Raw"

output_yc[4+n_methods*(i-1), 2] = fit_yc$coefficients[1]

output_yc[4+n_methods*(i-1), 3:4] = confint.default(fit_yc)[1, 1:2]

}

output_ya_sc1 = cbind(output_ya, true_ya)

output_yb_sc1 = cbind(output_yb, true_yb)

output_yc_sc1 = cbind(output_yc, true_yc)

save(output_ya_sc1,file="output_ya_sc1.R")

save(output_yb_sc1,file="output_yb_sc1.R")

save(output_yc_sc1,file="output_yc_sc1.R")

#---------------------------------

#Step 4: Plot and summary

#---------------------------------

g_s1_cont <- ggplot(output_ya, aes(x=A0_est, color=Model)) +

geom_density(aes(color=Model), size=1) +

ylab("Density")+xlab("Estimate of effect: mean difference") +

geom_vline(xintercept = true_ya)+theme_bw() +

scale_x_continuous(limits=c(1.5,6)) +

scale_color_manual(values=c("red", "green", "blue", "purple", "yellow3", "black"))

g_s1_bin <- ggplot(output_yb, aes(x=A0_est, color=Model)) +

geom_density(aes(color=Model), size=1) +

ylab("Density")+xlab("Estimate of effect: log(odds ratio)") +

geom_vline(xintercept = true_yb)+theme_bw() +

geom_vline(xintercept = 0, colour="gray40", linetype="dashed")+theme_bw() +

scale_x_continuous(limits=c(0,2.5)) +

scale_color_manual(values=c("red", "green", "blue", "purple", "yellow3", "black"))

g_s1_surv <- ggplot(output_yc[output_yc$Model!="MLR",], aes(x=A0_est, color=Model)) +

geom_density(aes(color=Model), size=1) +

ylab("Density")+xlab("Estimate of effect: log(hazard ratio)") +

geom_vline(xintercept = true_yc)+theme_bw() +

geom_vline(xintercept = 0, colour="gray40", linetype="dashed")+theme_bw() +

scale_x_continuous(limits=c(-2.0, 0)) +

scale_color_manual(values=c("red", "green", "blue", "purple", "yellow3", "black"))

save(g_s1_cont,file="g_s1_cont.r")

save(g_s1_bin,file="g_s1_cont.r")

save(g_s1_surv,file="g_s1_cont.r")

lay <- t(matrix(c(1,1,1,2,2,2,4,4,3,3,5,5),6,2))

grid.arrange(grobs=list(g_s1_cont,g_s1_bin,g_s1_surv),layout_matrix=lay,widths=c(0.1,0.1,0.2,0.2,0.1,0.1))

ggsave(file="scen1.png")

#Summarize the simulation results

k_ya<- output_ya %>%

group_by(Model) %>%

do(calc_absolute(., estimates = A0_est, true_param = true_ya))

c_ya<- output_ya %>%

group_by(Model) %>%

do(calc_coverage(., lower_bound = A0_lower, upper_bound = A0_upper, true_param = true_ya))

kc_ya<- data.frame(Model=k_ya$Model, K=k_ya$K_absolute, bias=k_ya$bias,

var=k_ya$var, mse=k_ya$mse, rmse=k_ya$rmse, coverage=c_ya$coverage, width=c_ya$width)

kc_ya_sc1_cont<-kc_ya %>%

kable(digits =5, format="simple") #create a kable table

k_yb<- output_yb %>%

group_by(Model) %>%

do(calc_absolute(., estimates = A0_est, true_param = true_yb))

c_yb<- output_yb %>%

group_by(Model) %>%

do(calc_coverage(., lower_bound = A0_lower, upper_bound = A0_upper, true_param = true_yb))

kc_yb<- data.frame(Model=k_yb$Model, K=k_yb$K_absolute, bias=k_yb$bias,

var=k_yb$var, mse=k_yb$mse, rmse=k_yb$rmse, coverage=c_yb$coverage, width=c_yb$width)

kc_ya_sc1_bin<-kc_yb %>%

kable(digits =5, format="simple") #create a kable table

k_yc<- output_yc %>%

group_by(Model) %>%

do(calc_absolute(., estimates = A0_est, true_param = true_yc))

c_yc<- output_yc %>%

group_by(Model) %>%

do(calc_coverage(., lower_bound = A0_lower, upper_bound = A0_upper, true_param = true_yc))

kc_yc<- data.frame(Model=k_yc$Model, K=k_yc$K_absolute, bias=k_yc$bias,

var=k_yc$var, mse=k_yc$mse, rmse=k_yc$rmse, coverage=c_yc$coverage, width=c_yc$width)

kc_ya_sc1_surv <- kc_yc %>%

kable(digits =5, format="simple") #create a kable table

save(kc_ya_sc1_cont,file="kc_ya_sc1_cont.r")

save(kc_ya_sc1_bin,file="kc_ya_sc1_bin.r")

save(kc_ya_sc1_surv,file="kc_ya_sc1_surv.r")

#---------------------------------

#---------------------------------

#---------------------------------

### Supplementary Section 3: Example of the application of Model Estimated Controls using the ‘psc’ package

The following is a copy of the example available from the github repository (github.com/richjjackson/psc)

The goal of psc is to compare an observational dataset against a parametric model

## Installation

You can install the development version of psc from [GitHub](https://github.com/) with:

# install.packages("devtools")

devtools::install_github("richJJackson/psc")

## Example

This is a basic example which shows you how to solve a common problem:

library(psc)

library(survival)

## basic example code

### Load model

data("surv.mod")

### Load Data

data("data")

### Use 'pscfit' to compare

surv.psc <- pscfit(surv.mod,data)

You can use standard commands for getting a summary of your analysis…

summary(surv.psc)

#> Summary:

#>

#> 100 observations selected from the data cohort for comparison

#> CFM of type flexsurvreg identified

#> linear predictor succesfully obtained with median:

#> trt: 3.15

#> Average expected response:

#> trt: 9.1

#> Average observed response: 6.366

#>

#> Counterfactual Model (CFM):

#> A model of class 'flexsurvreg'

#> Fit with 3 internal knots

#>

#> Formula:

#> Surv(time, cen) ~ vi/age60 + ecog + allmets + logafp + alb +

#> logcreat + logast + aet

#> <environment: 0x11a95d660>

#>

#> Call:

#> CFM model + beta

#>

#> Coefficients:

#> median 2.5% 97.5% Pr(x<0) Pr(x>0)

#> beta 0.3574 0.1204 0.5801 0.0004 0.9996

#> DIC 280.8601 273.6541 291.9444 NA NA

… and to see a plot of what you have done

[
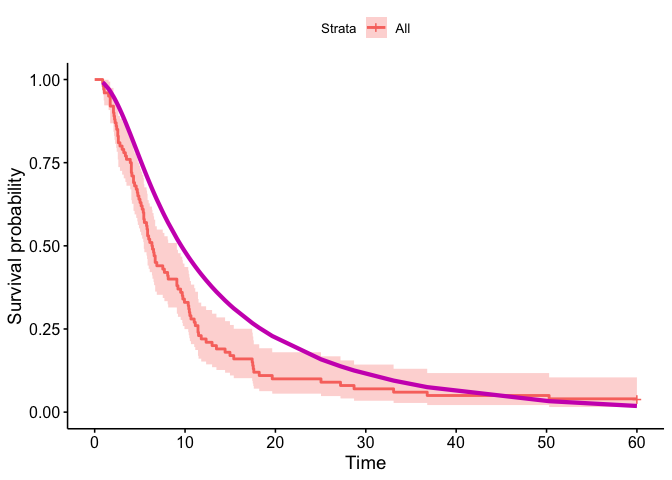
](https://github.com/richJJackson/psc/blob/main/man/figures/README-pressure-1.png)
